# Stereotactic ablative radiotherapy for the comprehensive treatment of 1–3 Oligometastatic tumors (SABR-COMET-3): study protocol for a randomized phase III trial

**DOI:** 10.1186/s12885-020-06876-4

**Published:** 2020-05-05

**Authors:** Robert Olson, Lindsay Mathews, Mitchell Liu, Devin Schellenberg, Benjamin Mou, Tanya Berrang, Stephen Harrow, Rohann J. M. Correa, Vasudeva Bhat, Howard Pai, Islam Mohamed, Stacy Miller, Famke Schneiders, Joanna Laba, Derek Wilke, Sashendra Senthi, Alexander V. Louie, Anand Swaminath, Anthony Chalmers, Stewart Gaede, Andrew Warner, Tanja D. de Gruijl, Alison Allan, David A. Palma

**Affiliations:** 1 Department of Radiation Oncology, BC Cancer – Centre for the North, 1215 Lethbridge Street, Prince George, British Columbia V2M7E9 Canada; 2 BC Cancer – Vancouver, Vancouver, British Columbia Canada; 3 BC Cancer – Surrey, Surrey, British Columbia Canada; 4 BC Cancer – Kelowna, British Columbia, Kelowna, Canada; 5 BC Cancer – Victoria, Victoria, British Columbia Canada; 6 Beatson West of Scotland Cancer Centre, Glasgow, Scotland; 7 Department of Oncology, London Health Sciences Centre, London, Ontario Canada; 8 Department of Radiation Oncology, Amsterdam University Medical Centre (UMC), Vrije Universiteit Amsterdam, Cancer Center Amsterdam, Amsterdam, The Netherlands; 9 Nova Scotia Cancer Centre, Halifax, Nova Scotia Canada; 10 Alfred Health Radiation Oncology, Melbourne, Australia; 11 Department of Radiation Oncology, Odette Cancer Centre, Sunnybrook Health Sciences Centre, Toronto, Ontario Canada; 12 University of Glasgow, Glasgow, Scotland; 13 Department of Oncology, Amsterdam UMC, Vrije Universiteit Amsterdam, Cancer Center Amsterdam, Amsterdam, the Netherlands

**Keywords:** Oligometastases, Stereotactic radiotherapy, Quality of life, Cancer, Survival

## Abstract

**Background:**

A recent randomized phase II trial evaluated stereotactic ablative radiotherapy (SABR) in a group of patients with a small burden of oligometastatic disease (mostly with 1–3 metastatic lesions), and found that SABR was associated with a significant improvement in progression-free survival and a trend to an overall survival benefit, supporting progression to phase III randomized trials.

**Methods:**

Two hundred and ninety-seven patients will be randomized in a 1:2 ratio between the control arm (consisting of standard of care [SOC] palliative-intent treatments), and the SABR arm (consisting of SOC treatment + SABR to all sites of known disease). Randomization will be stratified by two factors: histology (prostate, breast, or renal vs. all others), and disease-free interval (defined as time from diagnosis of primary tumor until first detection of the metastases being treated on this trial; divided as ≤2 vs. > 2 years). The primary endpoint is overall survival, and secondary endpoints include progression-free survival, cost effectiveness, time to development of new metastatic lesions, quality of life (QoL), and toxicity. Translational endpoints include assessment of circulating tumor cells, cell-free DNA, and tumor tissue as prognostic and predictive markers, including assessment of immunological predictors of response and long-term survival.

**Discussion:**

This study will provide an assessment of the impact of SABR on survival, QoL, and cost effectiveness to determine if long-term survival can be achieved for selected patients with 1–3 oligometastatic lesions.

**Trial registration:**

Clinicaltrials.gov identifier: NCT03862911. Date of registration: March 5, 2019,

## Background

Oligometastatic disease refers to a stage where a cancer has spread beyond the site of the primary tumor, usually limited to 1–3 or 1–5 sites, but is not yet widely metastatic [1]. In such patients, emerging evidence suggests that treatment of all sites of disease with ablative therapies (such as surgery or stereotactic ablative radiotherapy [SABR]) can improve patient outcomes, though an overall survival (OS) benefit has not been demonstrated in the setting of a phase III randomized trial.

To date, evidence to support the oligometastatic state has consisted of single-arm, non-randomized studies without controls, with OS estimates of 30–50% at 5 years [2, 3]. It is plausible that these reported long term survival estimates are mostly a result of selection bias [4, 5]. However, emerging phase II trials now provide some supporting evidence of an oligometastatic state, though phase III trial data is lacking, which has been outlined in the SABR-COMET-10 trial protocol published previously in this journal [6].

Most pertinent to this current trial, the Stereotactic Ablative Radiotherapy for the Comprehensive Treatment of Oligometastatic Disease (SABR-COMET) trial enrolled 99 patients who had controlled primary solid tumors and up to 5 metastatic lesions (most were 1–3 metastases). Patients were randomized in a 1:2 ratio between standard of care (SOC) palliative treatments (Arm 1) vs. SOC + SABR to all sites of disease (Arm 2) [7, 8]. The primary endpoint was OS, and the trial employed a randomized phase II screening design, with an alpha of 0.20, in order to provide an initial comparison between arms. OS was 28 vs 41 months in Arm 1 vs 2 (*p* = 0.09). Progression-free survival (PFS) was 6 vs12 months in Arm 1 vs 2 (*p* = 0.001). The grade 2 or higher toxicity from SABR was 29%, though the rate of grade 5 toxicity was almost 5%.

The results of SABR-COMET met the primary endpoint, with a trend toward improved OS with SABR, and have informed the design of this phase III randomized trial. This phase III trial will focus specifically on patients with 1–3 metastases, which comprised 92% of the patients on the SABR-COMET trial, as there was reluctance to accrue patients with 4–5 metastases, and theoretically survival benefit is hypothesized to be greatest in those with 1–3 metastases. In contrast to the SABR-COMET phase II trial [7], our phase III trial incorporates stratification by histology and disease-free interval instead of number of metastases.

## Methods/design

The objective of this trial is to assess the impact of SABR, compared SOC, on OS, oncologic outcomes, cost effectiveness, and QoL in patients with a controlled primary tumor and 1–3 metastatic lesions. See Appendix 1 for World Health Organization Trial Registration Dataset. The methods of this trial are similar to the sister trial SABR-COMET-10, as published elsewhere [6].

### Primary endpoint

#### OS

- Defined as time from randomization to death from any cause

### Secondary endpoints

#### PFS

- Defined as time from randomization to disease progression at any site or death from any cause, whichever occurs first

#### Time to development of new metastatic lesions

- Defined as time from randomization to development of new metastatic lesions, treating death from any cause as a competing event

#### Cost effectiveness

- The EuroQol 5-dimension 5-level (EQ-5D-5 L) questionnaire

#### QoL

- Assessed with the Functional Assessment of Cancer Therapy: General (FACT-G), site specific FACT subscales (e.g. FACT-Lung for chest metastases, FACT-Abdominal for adrenal metastases), and the EuroQol 5-Dimension 5-Level (EQ-5D-5 L)

#### Toxicity

- Assessed by the National Cancer Institute Common Toxicity Criteria (NCI-CTC) version 5 for each organ treated (e.g. liver, lung, bone)]

### Translational endpoints

- Assessment of circulating tumor cells (CTCs), cell-free DNA, and tumor DNA as prognostic and predictive markers of survival, and for early detection of progression
- Assessment of immunological predictors of response and long-term survival

## Study design

**Fig. 1:**
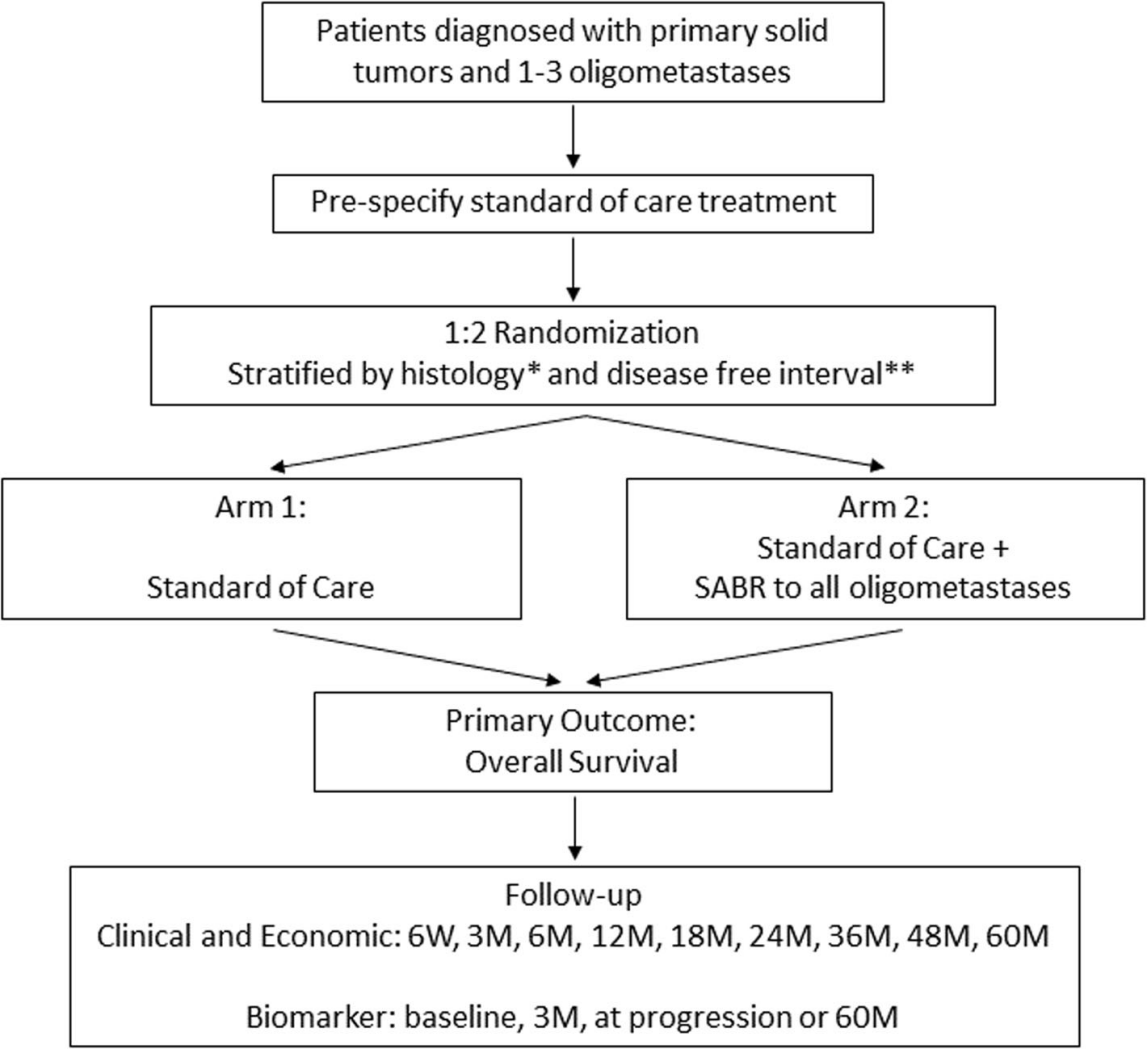
Study Schema. SABR = stereotactic ablative radiotherapy; W = weeks; M = months. *histology dichotomized as prostrate, breast, or renal vs. all others. **disease free interval defined as time from diagnosis of primary tumor until first detection of metastatses, and dichotomized as ≤2 vs. > 2 years

This is a phase III multicentre randomized trial. Participating centres will be tertiary, academic hospitals or radiotherapy (RT) treatment centres in Canada, the United Kingdom, the Netherlands, and Australia (updated country list available on ClinialTrials.gov entry NCT03862911). Patients will be randomized in a 1:2 ratio between current SOC treatment (Arm 1) vs. SOC treatment + SABR (Arm 2) to sites of known disease (Fig. 1).

Patients will be stratified by (1) histology (prostate, breast, or renal vs. all others), and (2) disease free interval (defined as time from diagnosis of primary tumor until first detection of the metastases being treated on this trial; divided as ≤2 vs. > 2 years).

## Inclusion criteria

- Total number of metastases of 1–3
- Age 18 years or older
- Willing to provide informed consent
- ECOG performance status 0–2
- Life expectancy > 6 months
- Histologically confirmed malignancy with metastatic disease detected on imaging. Biopsy of metastasis is preferred, but not required.
- Controlled primary tumor
- defined as: at least 3 months since original tumor treated definitively, with no progression at primary site
- Previous systemic and radiation therapy is permitted
- Hormonal therapy is permitted
- A history and physical exam including performance status performed within 6 weeks of study accrual
- Not suitable for resection at all sites or decline surgery
- Patient has had a CT chest, abdomen and pelvis or PET-CT within 8 weeks of enrollment, and within 12 weeks of treatment
- Patient has had a nuclear bone scan (if no PET-CT) within 8 weeks of enrollment, and within 12 weeks of treatment
- If solitary lung nodule for which biopsy is unsuccessful or not possible, patient has had an 18-Fluorodeoxyglucose (18-FDG) Positron Emmision Tomography (PET) scan or CT (chest, abdomen, pelvis) and bone scan within 8 weeks of enrollment, and within 12 weeks of treatment
- If colorectal primary with rising carcinoembryonic antigen (CEA), but equivocal imaging, patient has had an FDG PET scan within 8 weeks of enrollment, and within 12 weeks of treatment
- Patient has had CT or MRI brain imaging if primary has a propensity for central nervous system metastasis within 8 weeks of enrollment, and within 12 weeks of treatment
- Patient is judged able to:
  - Maintain a stable position during therapy
  - Tolerate immobilization device(s) that may be required to deliver SABR safely
- Negative pregnancy test for Women of Child-Bearing potential (WOCB) within 2 weeks of RT start date
- Patient is able and willing to complete the QoL questionnaires, and other assessments that are a part of this study, via paper or online using REDCap (if email is provided by participant on informed consent)

## Exclusion criteria

- Serious medical comorbidities precluding RT. These include interstitial lung disease in patients requiring thoracic radiation, Crohn’s disease in patients where the gastrointestinal tract will receive RT, and connective tissue disorders such as lupus or scleroderma.
- No chemotherapy agents (cytotoxic, or molecularly targeted agents) are allowed within the period of time commencing 2 weeks prior to radiation, lasting until 1 week after the last fraction.
- Use of chemotherapy schemes containing potent enhancers of radiation damage (e.g. gemcitabine, adriamycin) are discouraged within the first month after radiation.
- Substantial overlap with a previously treated radiation volume. Prior RT in general is allowed, as long as the composite plan meets dose constraints herein. For patients treated with conventional radiation previously, biological effective dose calculations should be used to equate previous doses to the tolerance doses listed below. All such cases should be discussed with one of the study Principal Investigators.
- Malignant pleural effusion
- Inability to treat all sites of disease
- Maximum size of 6 cm for lesions outside the brain, except:
- Bone metastases over 6 cm may be included, if in the opinion of the local PI it can be treated safely (e.g. rib, scapula, pelvis)
- Any brain metastasis > 3 cm in size or a total volume of brain metastases greater than 30 cc.
- Clinical or radiologic evidence of spinal cord compression, or epidural tumor within 2 mm of the spinal cord. Patients can be eligible if surgical resection has been performed, but the surgical site counts toward the total of up to 3 metastases.
- Dominant brain metastasis requiring surgical decompression
- Pregnant or breast-feeding women

## Pre-treatment EVALUATION

- History and Physical Examination within 6 weeks of study accrual
  - Including prior cancer therapies and cancer-specific concomitant medications (e.g. systemic therapy such as immunotherapy, hormone therapy and/or chemotherapy drugs and regular/supporting medications such as anti-emetics).
- Re-staging within 8 weeks prior to randomization, and within 12 weeks of treatment:
  - Brain: CT or MRI for tumor sites with propensity for brain metastasis. All patients with brain metastases at enrollment or previously require an MRI.
  - Body: 18-FDG PET/CT imaging is strongly recommended, except for tumors where FDG uptake is not expected (e.g. prostate, renal cell carcinoma). Prostate Specific Membrane Antigen (PSMA)-PET or choline-PET is recommended for prostate cancer. In situations where a PET scan is unavailable, or for tumors that do not take up radiotracer, CT neck/chest/abdomen/pelvis with bone scan are required
  - Spine: MRI is required for patients with vertebral or paraspinal metastases, though the MRI can be limited to the involved segment, including at least the involved vertebral body (ies) plus 2 vertebral bodies above and below, where applicable.
- Pregnancy test for women of child-bearing potential within 2 weeks of RT start date

### Defining the number of metastases

Patients are eligible if there are 1–3 metastatic lesions present, with each discrete lesion counted individually. For patients with lymph node metastases, each node is counted seperately. All known metastatic lesions must be targetable on planning CT.

Patients with prior metastases that have been treated with ablative therapies (e.g. SABR, surgery, radiofrequency ablation) are eligible, as long as those metastases are controlled on imaging. In that case, the previously treated lesions are counted toward the total of 3.

When patients have small indeterminate nodules (e.g. a 2 mm lung nodule) it can be difficult to determine whether these are benign or whether they represent metastasis. Any such lesion that is ‘new’ is automatically considered a metastasis unless there are > 2 months of documented stability without systemic therapy.

### Brain metastases at presentation

If a patient presents with 1–2 brain metastases and ablation of those metastases is deemed to be clinically required regardless of the treatment of extracranial metastases, ablative treatment is permitted to the brain metastases as long as at least one extracranial metastasis is present that can be randomized. Those treated metastases count within the total number of 3 lesions. The patient would then be randomized to treatment of the extracranial disease. For example, a patient with a solitary brain metastasis and two lung metastases could receive an ablative technique to the brain (e.g. surgery, stereotactic radiosurgery [SRS], or fractionated stereotactic radiotherapy [FSRT]), and then be randomized to SABR vs SOC for the two lung metastases.

### Patients already receiving systemic therapy

Prior systemic therapy is not a contraindication to enrollment. Systemic therapy may be continued if randomized to the standard arm. However, if randomized to the experimental arm, patients will receive SABR between cycles, and may require a short treatment break.

## Interventions

### Standard arm (arm 1)

Patients on the standard arm should only be offered RT for palliation as per principles of the individual institution.. Recommended dose fractionations in this arm will include 8 Gy in 1 fraction, 20 Gy in 5 fractions. SABR should not be offered in this arm..

Systemic therapy (cytotoxic, targeted, hormonal, or immunotherapy) or observation may be used in the standard arm. See section 6.3 for the timing of systemic therapy.

### Experimental arm (arm 2)

#### Dose/fractionation

Table 1 summarizes the dose and fractionations to be used. All doses are prescribed to the periphery of the planning target volume (PTV).

**Table 1:** Dose and fractionations by site with [secondary options in square brackets]

Tumor Location	Description		Total Dose (Gy)	Number of fractions	Dose per fraction (Gy)	Frequency
**Lung**	Tumors 5 cm or less surrounded by lung parenchyma		48 [54]	4 [3]	12 [18]	Daily, or Every second day
	Within 2 cm of mediastinum or brachial plexus		60	8	7.5	Daily
**Bone**	Any bone		35 Gy [24]	5 [2]	7 [12]	Daily
**Brain**	Stereotactic lesions (no whole brain RT)	< 2 cm	24	1	24	Once
		2–3 cm	18	1	18	Once
		3–4 cm	15	1	15	Once
	If whole brain treated, then simultaneous boost to each lesion		35Gy to metastases 20 Gy whole brain (Opt)	5	7 Gy to PTV	Daily
					4 Gy WBRT	
**Liver**			54 Gy	3	18	Every second day
**Adrenal**			40 Gy	5	8	daily
**Lymph Node/Soft Tissue**			40 Gy	5	8	daily

RT – radiotherapy; WBRT – whole brain radiotherapy

#### Immobilization

Treatment will be setup using reproducible positioning and verified using an on-line protocol for all patients in this study. Immobilization may include a custom immobilization device, such as thermoplastic shell or vacuum bag, as per individual institutional practice when delivering SABR. Some centers do not use immobilization devices and have demonstrated high degrees of accuracy; this is acceptable in this study.

#### Imaging/localization/registration

All patients in Arm 2 will undergo planning CT simulation. 4-dimensional CT (4D-CT) will be used for tumors in the lungs, liver, or adrenals. Axial CT images will be obtained throughout the region of interest. For centres using SRS platforms, real-time tumor tracking and orthogonal imaging systems are permitted.

#### 4D-CT procedures

For patients undergoing 4D-CT, physics will review the 4D-CT images and will perform the following quality assurance procedures indicated on the 4D-CT template designed specifically for SABR:

i) Ensure all end inspiration (0%) tags exist and are in the right position. This ensures image integrity.
ii) If the quality of the 4D-CT images is not sufficient (determined by physics), then standard 3D-CT will be performed on the fast-helical CT or Untagged Average CT.
iii) Motion measurements in all 3 directions are performed:

1. If the motion is less than or equal to 7 mm and good quality images exist, then treatment planning may be performed on the Untagged Average CT with the 50% or 60% phase (End Expiration) and the 0% phase being fused to it. This will define the internal gross tumor volume (IGTV).
2. If the motion is greater than 7 mm in any one direction, then respiratory-gated RT can be considered. In this case, treatment planning will be performed on a subset average CT dataset (usually labeled either 30–60% Avg CT or 40–70% Avg CT) generated by Physics. This is an average CT over the intended gated interval. Therefore, the gross tumor volume (GTV) that is delineated on this scan will incorporate residual motion in the intended gated interval. The 0% phase will also be fused to this dataset. The PTV for planning will include the GTV delineated on the subset average CT plus margins for microscopic extension (at physician’s discretion) and setup uncertainty. The GTV_0% should also be delineated and combined with the GTV delineated on the subset average CT to define an additional volume labeled IGTV_CBCT. This contour may be used for image registration with cone beam CT (CBCT) only.

### Volume definitions (arm 2)

For all lesions, the GTV will be defined as the visible tumor on CT, MRI and/or PET imaging. No additional margin will be added for microscopic spread of disease (i.e. Clinical Target Volume [CTV] = GTV). For bone lesions, CTV of 3-5 mm will be allowed. For vertebral lesions, anatomic approach will be taken as per the International Spinal consortium guideline [9].

An anatomic approach is taken to the CTV based on where the disease within the spinal segment is located. The rules for CTV are as follows:

1. If the vertebral body is involved with GTV then the entire vertebral body is taken as CTV.
2. If the ipsilateral pedicle and/or transverse process have GTV then the entire ipsilateral posterior segment (pedicle, lamina and transverse process) ± the spinous process is taken into the CTV. The inclusion of the spinous process is per the discretion of the radiation oncologist.
3. If the ipsilateral pedicle, lamina, and/or transverse process have GTV, then the entire ipsilateral posterior segment (pedicle, lamina, and transverse process) +/− the spinous process is taken into the CTV.
4. If bilateral involvement of the pedicle and/or transverse process with GTV, then the posterior segment anatomy ± the spinous process is taken into the CTV. The inclusion of the spinous process is per the discretion of the radiation oncologist.
5. If bilateral involvement of the pedicles and lamina, and/or transverse process with GTV, then the entire posterior segment anatomy is taken into the CTV, including the spinous process.
6. If the spinous process is involved with GTV alone then the bilateral lamina ± pedicles are to be taken into the CTV.

The International Spinal Consortium Guideline is a reference for CTV delineation and can be adhered to as described (See Appendix 2) [9].

In the case of epidural disease, a 5 mm anatomic margin (excluding the spinal cord) beyond the GTV may be used within the epidural compartment including in the cranio-caudal direction. A circumferential CTV as per a donut based CTV is allowed and encouraged in the case of epidural disease at the discretion of the treating radiation oncologist. If paraspinal disease is present, a minimum 5 mm CTV margin may be applied beyond the GTV.

A PTV margin of 2–5 mm will be added depending on site of disease, immobilization, and institutional set-up accuracy: 2–3 mm margins should be used for spinal stereotactic treatments, 0–2 mm for brain tumors, and 5 mm for other sites.

Targets should be named based on the organ involved, and numbered from cranially to caudally. For example, in a patient with 3 lung lesions, there would be: GTV_lung_1, GTV_lung_2, and GTV_lung_3, and corresponding PTV_lung_1, PTV_lung_2, and PTV_lung_3, representing the lesions from superior to inferior.

For spinal lesions, a pre-treatment MRI is required to assess the extent of disease and position of the spinal cord. This must be fused with the planning CT scan. A Planning Organ at Risk Volume (PRV) expansion of 2 mm will be added to the spinal cord, and dose constraints for the spinal cord apply to this PRV. Alternatively, the thecal sac may be used as the PRV. For radiosurgery platforms, a PRV margin of 1 mm is permitted for the spinal cord.

#### Organ at risk (OAR) doses

OAR doses are listed in Appendix 2. OAR doses may not be exceeded except in the case of chest wall or ribs. In cases where the PTV coverage cannot be achieved without exceeding OAR doses, the PTV coverage is to be compromised. All serial organised OARs within 5 cm of the PTV must be contoured (partial organ contours allowed); for parallel organised organs (liver, lung, etc.) within 5 cm of PTV, the whole organs need to be contoured. This should be tested for each PTV by creating a 5 cm expansion to examine which OARs lie within that expansion.

#### Treatment planning

Treatment can be delivered using static beams (either 3D-conformal RT or intensity-modulated) or rotational therapy (volumetric modulated arc therapy [VMAT], or tomotherapy).

Dose constraints may not be exceeded (except chest wall or ribs). If a dose constraint cannot be achieved due to overlap of the target with an OAR, the fractionation can be increased or the target coverage compromised in order to meet the constraint. In cases where the target coverage or dose must be reduced, the priority for dose coverage is the GTV (e.g. attempt to cover as much of the GTV as possible with the prescription dose). All such cases of dose reduction or target coverage compromise must be approved by the local PI prior to treatment. For vertebral tumors, note that the spinal cord constraints apply to the PRV (see section 6.2.5).

For all targets, doses should be prescribed to 60–90% isodose line surrounding the PTV, and all hotspots should fall within the GTV. 95% of the PTV should be covered by the prescription dose, and 99% of the PTV should be covered by 90% of the prescription dose.

Doses must be corrected for tissue inhomogeneities. Several non-overlapping 6/10 MV beams (on the order of 7–11 beams) or 1–2 VMAT arcs combined possibly with a few non-coplanar beams should be utilized. Non-coplanar beams can be used to reduce 50% isodose volume.

The number of isocentres is at the discretion of the treating physician, physicists, and dosimetrists. Generally, metastases can be treated with separate isocenters if they are well-separated.

The scheduling and sequence of treating each metastasis is at the discretion of individual physicians, but in general should begin with the brain, due to risks associated with progression. Radiation schedule will depend on sites of tumor being treated, but generally daily or every other day for 1–3 weeks.

### Quality assurance (arm 2)

In order to ensure patient safety and effective treatment delivery, a robust quality assurance protocol is incorporated. The following requirements must be completed for each patient:

- Prior to treatment, each patient must be discussed at quality assurance rounds or be peer reviewed by a radiation oncologist with SABR expertise.
- All RT plans must meet target dose levels for OARs (except chest wall / ribs) (Appendix 2). Prior to plan approval, the dose to each OAR must be verified by the physicist or treating physician.
- All dose delivery for intensity-modulated plans (including arc-based treatments) will be confirmed before treatment by physics staff.

#### Systemic therapy

Patients treated with prior systemic therapy are eligible for this study, however, no chemotherapy agents (cytotoxic, immunotherapeutic, or molecularly targeted agents) are allowed within the period of time commencing 2 weeks prior to radiation lasting until 1 week after the last fraction. Hormonal therapy is allowed. Use of chemotherapy schemes containing potent enhancers of radiation damage (e.g. gemcitabine, Adriamycin, bevacizumab) are discouraged within the first month after radiation.

#### Further RT for progressive disease at new metastatic sites

Patients in Arm 1 who develop new metastatic deposits should not be treated with SABR, but rather be treated with standard of care approaches, such as systemic therapy or palliative RT.

Patients in Arm 2 who develop new, untreated metastatic deposits should be considered for SABR at those sites, if such deposits can be treated safely with SABR, and if the treating institution offers SABR for that body site. If SABR is not possible, then palliative RT can be delivered if indicated, as can systemic therapy.

#### Quality Assurance for Centres Joining Study

Each participating centre that was not involved in the original SABR-COMET study will be required to send to one of the Principal Investigators a mock treatment plan for the anatomic sites that will be treated (e.g. lung, brain, liver, adrenal), as outlined in the sister SABR-COMET-10 protocol [6].

## Subject discontinuation / withdrawal

Patients may discontinue participation in the study at any time. The clinical and laboratory evaluations that would have been performed at the end of the study should be obtained. If a subject is removed because of an adverse event, they should remain under medical observation as long as deemed appropriate by the treating physician.

Subjects withdrawn or discontinued can be replaced at the discretion of the Study Principal Investigator.

## Follow-up evaluation and assessment of efficacy

### Follow-up prior to progression

Patients will be seen at least every 6 months after treatment for 5 years. At each visit, a history and physical examination will be conducted by the oncologist or a delegated family physician (e.g. if patient is followed over video-link), and NCI-CTC toxicities recorded. The FACT-G, site-specific FACT subscales, and EQ-5D-5 L QoL instruments, and resource utilization questionnaires are to be completed at each visit, remotely (e.g. by phone, videolink, or mail), or the patient can complete these questionnaires at home (online using REDCap or on paper and mailed to the treating investigator).

CT head (or MR head), CT chest, abdomen and pelvis, bone scan will be repeated every 6 months, (+/− PET-CT, PSMA-PET as clinically indicated), for the first 2 years, then every 12 months until 5 years have elapsed. Head imaging can be omitted for histologies without a propensity for brain metastases (e.g. prostate). PET-CT scanning may be used in follow-up for patients who were staged with a PET-CT scan for trial entry. In such cases, the PET-CT replaces the CTs of the chest, abdomen, pelvis and the bone scan; brain imaging would still be required for histologies with a propensity for brain metastases (as defined by investigator). Patients with prostate cancer who have a prostate specific antigen (PSA) below 5 ng/mL may omit imaging requirements.

Since many patients will be receiving systemic therapy and separately-timed imaging may be required to assess response, attempts should be made to avoid duplication of scans. The imaging requirements herein may be adjusted by +/− 6 weeks, from target follow-up date, in order to align with scans used to assess response to systemic therapy (see Table 2).

**Table 2:** SABR-COMET-3 Follow-up Evaluation and Assessment of Efficac

Assessment	Screening Baseline	Enrollment (Day 0)	Treatment Visit 2 (Day 14)	Follow-up visit								
				6 W	3 M	6 M (±6 W)	12 M (±6 W)	18 M (±6 W)	24 M (±6 W)	36 M (±6 W)	48 M (±6 W)	60 M* (±6 W)
Informed Consent	X											
Inclusion/Exclusion	X											
Medical History	X											
Physical Exam	X											
**CT scans	X											
***Bone Scan or PET	X				Opt	Opt	Opt	Opt	Opt	Opt	Opt	Opt
Bloodwork & PFTs as applicable	X											
Pregnancy Test (Urinalysis)	X											
Randomization		X										
Medications		X		X	X	X	X	X	X	X	X	X
QoL questionnaires		X		X	X	X	X	X	X	X	X	X
CT Planning		X										
Quality Assurance		X										
SABR Treatment			X									
Toxicity Assessment				X	X	X	X	X	X	X	X	X
Adverse Events			X	X	X	X	X	X	X	X	X	X
EQ-5D-5 L		X			X	X	X	X	X	X	X	X
Resource Utilization (Patient and Provider Reported)					X	X	X	X	X	X	X	X

Footnotes: *W* weeks, *M* Months

*or early termination

**Extra imaging outside of study schedule is allowed per discretion of the study doctor

***Either bone or PET is required. If PET is done, bone scan is not required (or vice versa)

**/***Imaging is optional for prostate cancer patients with PSA < 5

### Follow-up after progression

After progression, patients randomized to Arm 2 will be considered for salvage SABR if new sites of disease develop, as long as it can be delivered safely, and to a maximum of 3 lesions total (including lesions treated at baseline).

After progression, additional visits, imaging or laboratory investigations should be carried out at the discretion of the oncologist. Additional treatment (e.g. further chemotherapy) is at the discretion of the oncologists. However, vital status and quality of life should still be collected, and this may be done remotely (e.g. by phone or mail) to minimize visit burden for patients.

## Translational biomarker studies

### Rationale

**Fig. 2:**
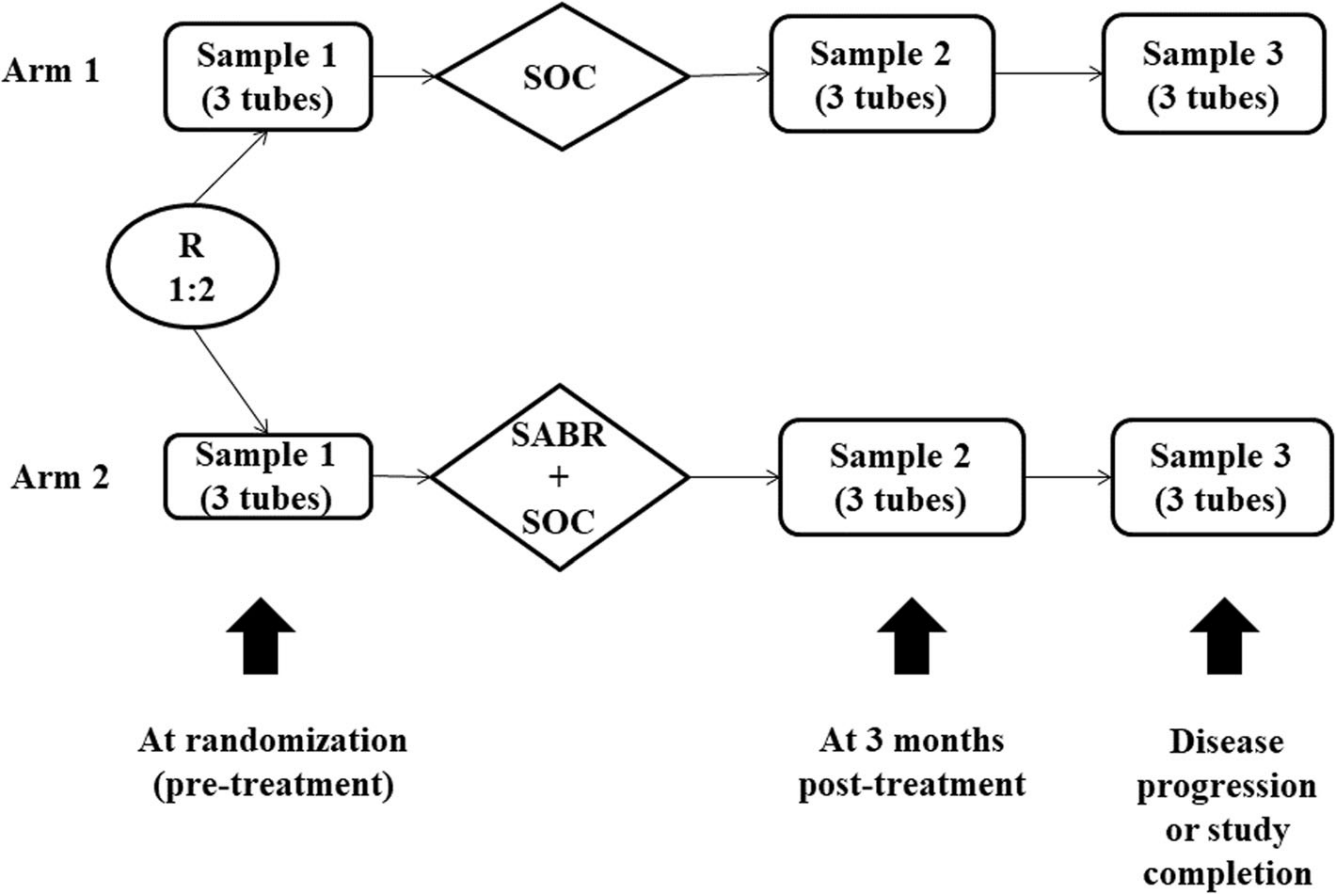
Peripheral Blood Collection Timeline. SOC = standard of care; SABR = stereotactic ablative radiotherapy. Study completion is defined as 5 years of follow-up. Samples will include 2 vials of blood for circulating tumor DNA and peripheral blood mononuclear cell isolation

The rational for the translational component is outlined in detail in the SABR-COMET-10 trial protocol published in this journal previously [6], which we will not reiterate here, and is summarized in Fig. 2. In brief, we will evaluate potential biomarkers though the use of a “liquid biopsy”, sampling peripheral blood to isolate and characterize biomarkers including circulating tumor DNA (ctDNA), CTCs, and/or circulating host immune cells, among others [10]. Liquid biopsy is an ideal sampling technique in this clinical trial because biopsy of metastatic lesions is not always possible.

## Statistical considerations

### Randomization

The study will employ a 1:2 randomization between Arm 1 and Arm 2, based on the stratification factors described in section 2. Patients will be randomized in permuted blocks, with the size of the blocks known only to the statistician and uploaded into a restricted-access database (REDCap) housed on secure hospital servers at BC Cancer. For each patient enrollment, the database will be accessed by the coordinating centre to obtain the next intervention in the random sequence, from the applicable stratum, to be assigned to the patient.

### Sample size calculation

The results of the original SABR-COMET phase II trial demonstrated a median OS of 28 months in the standard arm and 41 months in the experimental arm and a 22% improvement in 5-year OS. Based on these results, this phase III trial will aim to detect a hazard ratio (HR) of death of 0.66 in the experimental arm compared to the standard arm (equivalent to a 40% reduction in the hazard rate of death). Based on a 5-year OS of 20% for the standard arm, a HR of 0.66 represents a 15% improvement in OS, smaller than the effect seen in the phase II trial. In order to detect this difference, with 80% power, alpha of 0.05, an 8% dropout rate, accrual time of 5 years and a total trial time of 8 years, 297 patients will be required (99 patients in Arm 1 and 198 patients in Arm 2).

### Analysis plan

Patients will be analyzed in the groups to which they are assigned (intention-to-treat). De-identified data (except for study number and initials, see confidentiality below) will be transmitted from participating centres via REDCap to be collected centrally where it will be stored on secure hospital servers at LHSC. Source documents will also be uploaded. Research coordinators (clinical trials staff) will perform data checks throughout the trial period will call participating centres or visit as necessary.

### Survival endpoints

OS and PFS will be calculated using the Kaplan-Meier method with differences compared using the stratified log-rank test. Time to development of new metastases will be estimated using the Kaplan-Meier method and cumulative incidence functions with death as competing event, and differences compared using the stratified Gray’s test. Pre-planned subgroup analysis will occur based on the stratification factors, and also based on the use of immunotherapy vs. non-immunotherapy systemic agents. Cox proportional hazards multivariable regression analysis will be used to determine baseline factors predictive of survival endpoints. For time to development of new metastases, a Fine and Gray competing risk analysis will be used to account for competing risk of death.

### Secondary endpoints

QoL at 6 months will be measured using FACT-G, site-specific FACT subscales and EQ-5D-5 L, with differences between groups tested using the two-sample t-test, Chi-square test or Fisher’s Exact Test, as appropriate. Differences in rates of grade 2 or higher toxicity between groups will be tested using the Chi-square test or Fisher’s Exact Test, as appropriate.

### Cost utility analysis (CUA)

A CUA will be conducted in accordance with the Canadian Agency for Drugs and Technologies in Health (CADTH) Guidelines for the Economic Evaluation of Health Technologies Non-parametric bootstrapping will be used to estimate the 95% confidence intervals and to construct a cost-effectiveness acceptability curve. Sensitivity analysis will be conducted by varying the major drivers of costs. All costs will be adjusted to a base year using the healthcare component of the Statistics Canada Consumer Price Index to adjust for price inflation over time. Subsequent incremental cost per unit of OS improvement using OS outcomes will be explored. Although Canada has a single-payer health insurance system, the provincial and territorial governments are responsible for health care administration and delivery. Our analyses will be undertaken from the perspectives of the British Columbia (BC) and Ontario provincial Ministries of Health as we expect these provinces to accrue the highest number of patients. We will gain consent from all trial participants to prospectively assess their patient-level records pertaining to the frequency of hospital admissions and the use of targeted- and immunotherapies. We will use the resource costing method whereby utilization data are collected from existing data sources and then multiplied by unit costs.

### Data safety monitoring committee and interim analyses

The data and safety monitoring committee (DSMC) will review blinded safety data once 50 patients are accrued, and every 6 months thereafter. There are two planned interim analyses for efficacy in addition to the final analysis. For each interim analysis, the DSMC will be blinded to the identity of each treatment arm, but OS data will be presented for each arm. The two interim analyses are expected to be carried out when the total number of observed study deaths reaches 40 and 65, respectively; the final analysis is expected to be carried out 3 years after the enrollment of the last patient. The DSMC will recommend stopping the trial at either of the interim analyses if there is an OS difference that is statistically significant with a threshold of *p* < 0.001 based on the stratified log-rank test.

### Future pooled analysis with SABR-COMET-10

A separate but similar phase III trial, but for patients 4–10 metastases, called SABR-COMET-10, is open and running in parrallel with this current trial [6]. Once both trials are complete, a separate pooled analysis, using individual patient data from both trials, will be conducted, with the primary endpoint of OS, and any of the secondary endpoints from either trial where data has been collected in both trials.

## Ethical considerations

The Principal Investigator will obtain ethical approval and clinical trial authorization by competent authorities according to local laws and regulations.

### Institutional review board (IRB) / research ethics board (REB)

The protocol (and any amendments), the informed consent form, and any other written information to be given to patients will be reviewed and approved by a properly constituted Institutional Review Board (IRB)/Research Ethics Board (REB), operating in accordance with the current federal regulations (e.g., Canadian Food and Drug Regulations (C.05.001); US Code of Federal Regulations (21CFR part 56)), ICH GCP and local regulatory requirements. A letter to the investigator documenting the date of the approval of the protocol and informed consent form will be obtained from the IRB/REB prior to initiating the study. Any institution opening this study will obtain REB IRB/REB approval prior to local initiation.

### Informed consent

The written informed consent form is to be provided to potential study patients should be approved by the IRB/REB and adhere to ICH GCP and the ethical principles that have their origin in the Declaration of Helsinki. The investigator is responsible for obtaining written informed consent from each patient, or if the patient is unable to provide informed consent, the patient’s legally acceptable representative, prior to beginning any study procedures and treatment(s). The investigator should inform the patient, or the patient’s legally acceptable representative, of all aspects of the study, including the potential risks and benefits involved. The patient should be given ample time and opportunity to ask questions prior to deciding about participating in the study and be informed that participation in the study is voluntary and that they are completely free to refuse to enter the study or to withdraw from it at any time, for any reason.

The informed consent must be signed and dated by the patient, or the patient’s legally acceptable representative, and by the person who conducted the informed consent discussion. A copy of the signed and dated written informed consent form should be given to the patient or the patient’s legally acceptable representative. The process of obtaining informed consent should be documented in the patient source documents.

### Confidentiality of patient records

The names and personal information of study participants will be held in strict confidence. All study records (case report forms, safety reports, correspondence, etc.) will only identify the patient by initials and the assigned study identification number. The investigator will maintain a confidential patient identification list (Master List) during the course of the study. Access to confidential information (i.e., source documents and patient records) is only permitted for direct patient management and for those involved in monitoring the conduct of the study (i.e., Sponsors, CRO’s, representatives of the IRB/REB, and regulatory agencies). The patient’s name will not be used in any public report of the study.

## Confidentiality

The names and personal information of study participants will be held in strict confidence. All study records (case report forms, safety reports, correspondence, etc.) will only identify the patient by initials and the assigned study identification number. The investigator will maintain a confidential patient identification list (Master List) during the course of the study. Access to confidential information (i.e., source documents and patient records) is only permitted for direct patient management and for those involved in monitoring the conduct of the study (i.e., Sponsors, CRO’s, representatives of the IRB/REB, and regulatory agencies). The patient’s name will not be used in any public report of the study.

## Data sharing statement

De-identified participant data from this trial will not be shared publicly, however, the full protocol will be published along with the primary analysis of the outcomes.

## Protocol AMMENDMENTS and trial publication

Any modifications to the trial protocol must be approved and enacted by the Principal Investigator (Current version: 4.0 on October 4, 2019). Protocol amendments will be communicated to all participating centres, investigators, IRBs, and trial registries by the principal investigator. Any communication or publication of trial results will be led by the principal investigator, and is expected to occur within 1 year of the primary analysis. Trial results will remain embargoed until conference presentation of an abstract or until information release is authorized. Authorship of the trial abstract and ultimately the full manuscript will be decided by the principal investigator at the time of submission. Professional writers will not be used for either abstract or manuscript preparation.

## Discussion

The oligometastatic paradigm hypothesizes the existence of an intermediate state between localized and widely-disseminated metastatic cancer [1]. Recent randomized data have helped to confirm the existence of the oligometastatic state and demonstrate that ablative therapy - including SABR - improves PFS and OS [7]. However, phase III randomized evidence is lacking, which this trial proposes to address.

The primary endpoint of SABR-COMET-3 is OS with secondary endpoints of PFS, cost effectiveness and QoL. Translational endpoints will also be assessed using peripheral blood samples collected at multiple time points to evaluate ctDNA, CTCs, and host immune cell activation. Thus, SABR-COMET-3 aims to determine both whether SABR improves OS in patients with 1–3 metastases as well as to identify biomarkers of oligometastasis that can help select those patients who are most likely to benefit.

## Supplementary information

**Additional file 1 Appendix 1**. World Health Organization Trial Registration Dataset. **Appendix 2**. Dose constraints for SABR arm

## Data Availability

Not applicable.

## Abbreviations

SABR: Stereotactic Ablative Radiotherapy
RT: Radiotherapy
OS: Overall Survival
PFS: Progression-Free Survival
SABR-COMET: Stereotactic Ablative Radiotherapy for the Comprehensive Treatment of Oligometastatic Disease
SOC: Standard of Care
FACT-G: Functional Assessment of Cancer Therapy: General
NCI-CTC: National Cancer Institute Common Toxicity Criteria
CT: Computed Tomography
MRI: Magnetic Resonance Imaging
18-FDG: 18-Fluorodeoxyglucose
PET: Positron Emisson Tomography
PSMA: Prostate Specific Membrane Antigen
CBCT: Cone Beam Computed Tomography
PTV: Planning Target Volume
4D-CT: Four-Dimensional Computed Tomography
GTV: Gross Tumour Volume
IGTV: Internal Gross Tumor Volume
CTV: Clinical Target Volume
PRV: Planning Organ at Risk Volume
VMAT: Volumetric Modulated Arc Therapy
QA: Quality Assurance
CTC-AE: Common Toxicty Criteria for Adverse Events
DSMC: Data Safety Monitoring Committee
CTC: Circulating Tumor Cells
ctDNA: Circulating Tumor Deoxyribonucleic Acid
