# Supplementary material for: Stereotactic ablative radiotherapy for the comprehensive treatment of 1–3 Oligometastatic tumors (SABR-COMET-3): study protocol for a randomized phase III trial
Source: BMC Cancer. 2020 May 5;20:380. doi: 10.1186/s12885-020-06876-4 (PMC7201684; doi:10.1186/s12885-020-06876-4)
Supplement: Supplementary file 1 — Additional file 1 Appendix 1. World Health Organization Trial Registration Dataset. Appendix 2. Dose constraints for SABR arm [file 12885_2020_6876_MOESM1_ESM.docx]

**Appendices**

# APPENDIX 1: World Health Organization Trial Registration Dataset

| **Item** | **Description** |
| --- | --- |
| Primary registry and trial identifying number | ClinicalTrials.gov NCT03862911 |
| Date of registration in primary registry | March 5, 2019 |
| Secondary identifying numbers | NA |
| Source(s) of monetary or material support | Varian Medical Systems  BC Cancer Foundation  Canadian Institutes for Health Research |
| Primary sponsor | BC Cancer - Dr. Robert Olson |
| Secondary sponsor(s) | NA |
| Contact for public queries | Dr. Robert Olson |
| Contact for scientific queries | Dr. Robert Olson |
| Public title | High-dose, precision radiation therapy to treat all sites of metastatic disease in patients with 1-3 metastases |
| Scientific title | Stereotactic Ablative Radiotherapy for Comprehensive Treatment of Oligometastatic (1-3 Metastases) Cancer (SABR-COMET-3) |
| Countries of recruitment | Canada, United Kingdom, Ireland, Australia |
| Health condition(s) or problem(s) studied | Metastatic cancer |
| Intervention(s) | Standard Arm: Standard of care treatment: palliative radiotherapy, chemotherapy, immunotherapy, hormones, or observation, is at the discretion of the treating oncologist. |
|  | Stereotactic Arm: Stereotactic ablative radiotherapy, plus standard of care treatment: chemotherapy, immunotherapy, hormones, or observation given at the discretion of the treating oncologist. |
| Key inclusion and exclusion criteria | - Inclusion: Total number of metastases of 1-3 - Age 18 or older - Willing to provide informed consent - ECOG score 0-2 - Life expectancy >6 months - Histologically confirmed malignancy with metastatic disease detected on imaging. Biopsy of metastasis is preferred, but not required. - Controlled primary tumor   - defined as: at least 3 months since original tumor treated definitively, with no progression at primary site - Not suitable for resection at all sites or decline surgery - Patient has had a CT chest, abdomen and pelvis or PET-CT within 8 weeks of enrollment, and with 12 weeks of treatment - Patient has had a nuclear bone scan (if no PET-CT) within 8 weeks of enrollment, and with 12 weeks of treatment - If solitary lung nodule for which biopsy is unsuccessful or not possible, patient has had an 18-FDG PET scan or CT (chest, abdomen, pelvis) and bone scan within 8 weeks of enrollment, and with 12 weeks of treatment - If colorectal primary with rising CEA, but equivocal imaging, patient has had an 18-FDG PET scan within 8 weeks of enrollment, and with 12 weeks of treatment - Patient has had CT or MRI brain imaging if primary has a propensity for CNS metastasis within 8 weeks of enrollment, and with 12 weeks of treatment - Patient is judged able to: - Maintain a stable position during therapy - Tolerate immobilization device(s) that may be required to deliver SABR safely - Negative pregnancy test for Women of Child-Bearing potential (WOCB) within 2 weeks of RT start date - Patient is able and willing to complete the quality of life questionnaires, and other assessments that are a part of this study, via paper or online using REDCap (if participant email is provided on the informed consent) |
|  | - Exclusion: Previous SABR to the lesion(s) - Lesion in femoral bone requiring surgical fixation - No chemotherapy agents (cytotoxic, or molecularly targeted agents) are allowed within the period of time commencing 2 weeks prior to radiation, lasting until 1 week after the last fraction. - Use of chemotherapy schemes containing potent enhancers of radiation damage (e.g. gemcitabine, adriamycin) are discouraged within the first month after radiation. - Serious medical comorbidities precluding radiotherapy. These include interstitial lung disease in patients requiring thoracic radiation, Crohn's disease in patients where the GI tract will receive radiotherapy, and connective tissue disorders such as lupus or scleroderma. - Substantial overlap with a previously treated radiation volume. Prior radiotherapy in general is allowed, as long as the composite plan meets dose constraints herein. For patients treated with conventional radiation previously, biological effective dose calculations should be used to equate previous doses to the tolerance doses listed below. All such cases should be discussed with one of the study PIs. - Malignant pleural effusion - Inability to treat all sites of disease - Maximum size of 6 cm for lesions outside the brain, except:   - Bone metastases over 6 cm may be included, if in the opinion of the local PI it can be treated safely (e.g. rib, scapula, pelvis)   - Any brain metastasis >3 cm in size or a total volume of brain metastases greater than 30 cc. - Clinical or radiologic evidence of spinal cord compression, or epidural tumor within <2 mm of the spinal cord. Patients can be eligible if surgical resection has been performed, but the surgical site counts toward the total of up to 3 metastases. - Dominant brain metastasis requiring surgical decompression - Pregnant or breast feeding women |
| Study type | Randomized by permuted blocks sequence |
|  | No masking/blinding (open label) |
|  | Parallel assignment |
|  |  |
| Date of first enrolment | November 1, 2019 |
| Target sample size | 297 |
| Recruitment status | Recruiting |
| Primary outcome(s) | Overall survival |
| Key secondary outcomes | Progression-free survival; Time from randomization to development of new metastasis; Quality of Life; Toxicity; Resource Utilization, Translational endpoints; |

# APPENDIX 2: Dose Constraints

These are based on the AAPM TG 101, SABR-COMET, SC-24 trials as well as most updated references. If any structure is not listed, the constraints may be calculated using the linear quadratic formula from accepted QUANTEC doses, using an alpha-beta ratio of 3 (except neural structure: alpha-beta of 2) for late effects.

| **OAR** | **SABR ORGAN-AT-RISK (OAR) CONSTRAINTS** | | | | | | | |
| --- | --- | --- | --- | --- | --- | --- | --- | --- |
|  | Dmax to ≤=0.035cc for all OARs.  Exception: Dmax to 0cc (hard max) 🡪 Spinal cord, Spinal cord PRV, Thecal sac/Cauda Equina | | | | | | | |
|  | **Fractions** | | | | | | | |
|  | **2** | **3** | **4** | | **5** | | **8** | |
| **Spinal Cord PRV  (2mm on cord)**  **(or Spinal Canal)** | Dmax ≤ 17 Gy^12^ | Dmax ≤ 20.3 Gy^13^ | Dmax ≤ 23 Gy ^13^ | | Dmax ≤ 25.3 Gy ^13^ | | Dmax ≤ 30.6 Gy^13^ | |
| **Spinal Cord PRV /Thecal Sac (reirradiation)** | See Table 6 below^11^  (NB: 1^st^ row with 0 nBED not applicable, see above for 1^st^ time spine treatment) | | | | | | | |
| **Thecal Sac /  Cauda equina** | Dmax ≤ 17 Gy^12^ | Dmax ≤ 24 Gy^1^  V21.9 Gy ≤ 5 cc^1^ | | D_max_ ≤ 29 Gy ^1*^  V27 Gy ≤ 5 cc^1*^ | | Dmax ≤ 32 Gy^1^  V30 Gy ≤ 5 cc^1^ | | Dmax ≤ 39 Gy^1*^  V36 Gy ≤ 5 cc^1*^ |
| **Sacral plexus** | Dmax ≤ 26 Gy^12^ (+ nerve roots) | Dmax ≤ 24 Gy^1^  V22.5 Gy ≤ 5 cc^1^ | | D_max_ ≤ 29 Gy ^1*^  V27 Gy ≤ 5 cc^1*^ | | Dmax ≤ 32 Gy^1^  V30 Gy ≤ 5 cc^1^ | | Dmax ≤ 39 Gy^1*^  V36 Gy ≤ 5 cc^1*^ |
| **Brainstem** | NA | (not medulla)  Dmax ≤ 23.1 Gy^1^  V18 Gy ≤ 0.5 cc^1^ | | (not medulla) Dmax ≤ 28 Gy^1*^  V21 Gy ≤ 0.5 cc^1*^ | | (not medulla)  Dmax ≤ 31 Gy ^1^  V23 Gy ≤ 0.5 cc^1^ | | NA |
| **Optic Pathway** | Dmax≤ 15.8 Gy^17^  D0.2cc≤ 11.5Gy ^17^ | Dmax ≤ 19.5 Gy^17^  D0.2cc ≤ 15.0 Gy^17^ | | Dmax ≤ 22.5 Gy^17^  D0.2cc ≤ 17.5Gy^17^ | | Dmax ≤ 25 Gy^17^  D0.2cc ≤ 20 Gy^17^ | | NA  NA |
| **Cochlea** | Dmax ≤ 16.5 Gy^18^ | Dmax ≤ 20 Gy^18^ | | Dmax ≤ 22.5 Gy^18^ | | Dmax ≤ 25.0 Gy^18^ | | NA |
| **Parotids (each)** | Mean ≤ 7 Gy^12^ | NA | | NA | | NA | | NA |
| **Pharynx** | Dmax ≤ 20Gy^12^ Mean ≤ 9Gy^12^ | NA | | NA | | NA | | NA |
| **Larynx** | Dmax ≤ 20Gy^12^ Mean ≤ 9Gy^12^ | NA | | NA | | NA | | NA |
| **PBT and PT  (prox. bronch tree & prox. trachea)** | Dmax ≤ 20 Gy^12^ | Dmax ≤ 30 Gy ^1^ | | Dmax ≤ 34.8 Gy^3^ | | Dmax ≤ 40 Gy^1^ | | Dmax ≤ 46.3 Gy^3*^ |
| **Lungs-GTV** | NA | >1500cc ≤ 11.6 Gy^1^  V18 Gy ≤ 10%^3,8*^  Mean ≤ 5.5 Gy ^8*^ | | >1500cc≤ 11.6 Gy^3^  V20 Gy ≤ 10%^3,8^  Mean ≤ 6 Gy ^8^ | | >1500cc ≤12.5 Gy^1^  V22 Gy ≤ 10%^3,8*^  Mean ≤ 6.5 Gy ^8*^ | | > 1500c ≤ 14 Gy^3*^  V26Gy ≤ 10%^3,8*^  Mean ≤ 7 Gy ^8*^ |

| **OAR** | **SABR ORGAN-AT-RISK (OAR) CONSTRAINTS** | | | | |
| --- | --- | --- | --- | --- | --- |
|  | Dmax to ≤=0.035cc for all OARs.  Exception: Dmax to 0cc (hard max) 🡪 Spinal cord, Spinal cord PRV, Thecal sac/Cauda Equina | | | | |
|  | **Fractions** | | | | |
|  | **2** | **3** | **4** | **5** | **8** |
| **Each Lung** | V5 Gy ≤ 35%^12^  V10 Gy ≤ 10%^12^  V20 Gy ≤ 3%^12^  Mean ≤ 5 Gy^12^ | NA | NA | NA | NA |
| **Chest wall and Ribs:** | Dmax ≤ 36.5 Gy^7*^  V25Gy < 30cc^6*^ | Dmax ≤ 44 Gy ^7^  V30 Gy ≤ 30 cc^6^ | Dmax ≤ 50 Gy ^7^  V34 Gy ≤ 30 cc^6^ | Dmax ≤ 55Gy ^7^  V37 Gy ≤ 30cc^6^ | Dmax ≤ 68 Gy^7^  V45 Gy ≤ 30 cc^6^ |
| **Brachial Plexus** | NA | Dmax ≤ 24 Gy ^1^  V20.4 Gy ≤ 3cc^1^ | Dmax ≤ 26 Gy ^4^  V23.6 Gy ≤ 3 cc ^3^ | Dmax ≤ 30.5 Gy ^1^  V27 Gy ≤ 3 cc^1^ | Dmax ≤ 35 Gy ^4^ |
| **Heart / Pericardium** | NA | Dmax ≤ 30 Gy^1^  V24 Gy ≤ 15 cc^1^ | Dmax ≤ 34 Gy ^3^  V28 Gy ≤ 15 cc ^3^ | Dmax ≤ 38 Gy^1^  V32 Gy ≤ 15 cc^1^ | Dmax ≤ 46 Gy ^2^  V39 Gy ≤ 15 cc ^2^ |
| **Great Vessels** | Dmax ≤ 40 Gy^20^ | Dmax ≤ 44 Gy^20^ | Dmax ≤ 49.0 Gy^20^ | Dmax ≤ 51.5 Gy^20^ | Dmax ≤ 65 Gy ^2^ |
| **Skin** | Dmax < 26 Gy^1*^  V24 Gy < 10 cc^1*^ | Dmax ≤ 33 Gy^1^  V30Gy ≤ 10 cc^1^ | Dmax ≤ 36 Gy ^3^  V33.2 Gy ≤ 10 cc ^3^ | Dmax ≤ 39.5 Gy^1^  V36.5 Gy ≤ 10 cc^1^ | Dmax ≤ 48 Gy^3*^  V44 Gy ≤ 10 cc^3*^ |
| **Esophagus** | Dmax ≤ 20 Gy^12^ | Dmax ≤ 27.0 Gy^21^ | Dmax ≤ 30 Gy ^3, 21^ | Dmax ≤ 35 Gy^1, 21^ | Dmax ≤ 40 Gy ^2^ |
| **Stomach** | Dmax ≤ 20 Gy^12^ | Dmax ≤ 22.2 Gy^1^  (Dmax ≤25 Gy  if PTV close by; MRP to specify^14^) | Dmax ≤ 27 ^3^ | Dmax ≤ 32 Gy^1^  (Dmax ≤ 35 Gy  if PTV close by ; MRP to specify^14^ ) | Dmax ≤ 40 Gy ^2^ |
| **Duodenum** | Dmax ≤ 20 Gy^12^ | Dmax ≤ 22.2 Gy^1^  (Dmax ≤25 Gy  if PTV close by ; MRP to specify^14^) | Dmax ≤ 29.0 Gy^1*^ | Dmax ≤ 32 Gy^1,22^  (Dmax ≤ 35 Gy if PTV close by ; MRP to specify^14^ ) | Dmax ≤ 39 Gy ^2^ |
| **Small Bowel**  **(Jejunum/ileum)** | Dmax ≤ 20 Gy^12^ | Dmax ≤ 25.2 Gy^1, 23^ | Dmax ≤ 28.5 Gy^23^ | Dmax ≤ 29.0 Gy^23^  (Dmax ≤ 35 Gy^1^  if PTV close by MRP to specify) | Dmax ≤ 40 Gy ^2^ |
| **Large Bowel**  **(Colon, Rectum)** | Dmax ≤ 20 Gy^12^ | Dmax ≤ 28.2 Gy ^1^ | D_max_ ≤ 34.5 Gy ^1*^ | Dmax ≤ 38 Gy ^1^ | Dmax ≤ 46 Gy^1^ |
| **Kidneys (each)** | Dmax ≤ 26 Gy^12^  Mean ≤ 6 Gy ^12^ (*each kidney) | NA | NA | NA | NA |
| **Renal Cortex (Kidneys)**  **(R & L combined)** | See  “Kidneys (each)” | > 200 cc ≤ 14.4 Gy^2^ | > 200 cc < 16.2Gy^1*^ | > 200cc ≤ 17.5 Gy^1^ | > 200 cc ≤ 21 Gy ^2^ |
| **Liver (Liver minus GTV)** | Dmax ≤ 26 Gy^12^  Mean ≤ 8 - 9 Gy^12^ | >700 cc ≤ 17 Gy^1^ | >700 cc ≤ 19 Gy ^1*^ | > 700 cc ≤ 21Gy^1^ | > 700 cc ≤ 22 Gy^2^ |
| **Bladder Wall** | NA | Dmax ≤ 28.2 Gy^1^ | D_max_ ≤ 34.5 Gy ^1*^ | Dmax ≤ 38 Gy^1^ | NA |

| **OAR** | **SABR ORGAN-AT-RISK (OAR) CONSTRAINTS** | | | | |
| --- | --- | --- | --- | --- | --- |
|  | Dmax to ≤=0.035cc for all OARs.  Exception: Dmax to 0cc (hard max) 🡪 Spinal cord, Spinal cord PRV, Thecal sac/Cauda Equina | | | | |
|  | **Fractions** | | | | |
|  | **2** | **3** | **4** | **5** | **8** |
| **Penile Bulb** | NA | Dmax ≤ 42 Gy ^1^  V21.9 Gy ≤ 3 cc^1^ | NA | Dmax ≤ 50 Gy^1^  V30 Gy ≤ 3 cc^1^ | NA |
| **Femoral Heads (R & L combined)** | NA | V21.9 Gy ≤ 10 cc^1^ | V27 Gy ≤ 10 cc ^1*^ | V30 Gy ≤ 10 cc^1^ | NA |

*Values are EQD2 conversion from stated reference


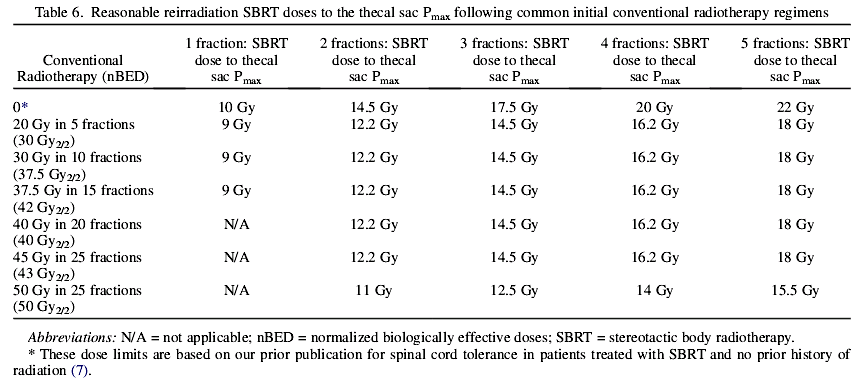


**References for Appendix 1**

^1^ Benedict SH et al., AAPM TG101, Med.Phys 37(9), 2010 (NB: max pt = 0.035cc)
^2^ Palma et al., SABR-COMET Trial, Palma et al, v.1.9, Apr 2015
^3^ RTOG 0915, <http://www.rtog.org/ClinicalTrials/ProtocolTable/StudyDetails.aspx?study=0915>.

^4^ Forquer JA et al., Radiother Onc, 93:408-413, 2009

^5^ Karlsson K et al., IJROBP, 87(3): 590-595, 2013

^6^ Dunlap NE et al., IJROBP 76(3): 796-801, 2010

^7^ Andolino DL et al., IJROBP 80(3): 692-697, 2011

^8^ Baker R, IJROBP, 85(1):190-195, 2013
^9^ Matsuo Yet al., IJROBP, 83(4): e545-549, 2012.
^10^ Bongers EM et al., Radiother Onc, 109: 95-99, 2013 (VU EMC TO 2013)
^11^ Sahgal A et al., IJROBP, 82(1):107-116, 2012
^12^ Sahgal et al., NCIC CTG Protocol #SC.24. Feb 2, 2017

^13^ Sahgal A et al., IJROBP, 85(2):341-7, 2013 ( Table 5; using 5% risk as per Sahgal ; personal communication Feb 2017 )

^14^ BCCA liver protocol 2011

^15^ Velec M, et al, IJROBP (2017), doi: 10.1016/j.ijrobp.2017.01.221
^16^ Grimm, et al, JACMP (2011), 12(2) 267

^17^Hiniker, et al., Sem Radiat Oncol 26(2), 97-104, 2016. DOI: (10.1016/j.semradonc.2015.11.008)

^18^Rashid, et al., Sem Radiati Oncol 26(2) 105-111, 2016. DOI: (10.1016/j.semradonc.2015.11.004)

^19^Kimsey et al., Sem Radiat Oncol 26(2), 129-134, 2016. DOI: (10.1016/j.semradonc.2015.11.003)

^20^Xue et al, Sem Radiat Oncol 26(2), 135-139, 2016. DOI: (10.1016/j.semradonc.2015.11.001)

^21^Nuyttens et al., Sem Radiat Oncol 26(2), 120-128, 2016. DOI: (10.1016/j.semradonc.2015.11.006)
^22^Goldsmith et al., Sem Radiat Oncol 26(2), 149-156, 2016. DOI: (10.1016/j.semradonc.2015.12.002)

^23^ LaCouture et al., Sem Radiat Oncol 26(2), 157-164, 2016. DOI: ([10.1016/j.semradonc.2015.11.009](https://doi.org/10.1016/j.semradonc.2015.11.009))

**Constraints for Dose Spillage and Conformality**

The R100 (ratio of size of prescription isodose volume to size of PTV) should be less than 1.2. Exceptions are allowed for small PTVs. The R50 (ratio of size of 50% prescription isodose volume to size of PTV) should be as low as possible and conform to the table below. Values for the maximum dose 2 cm or more away from the PTV (D2cm) is below.


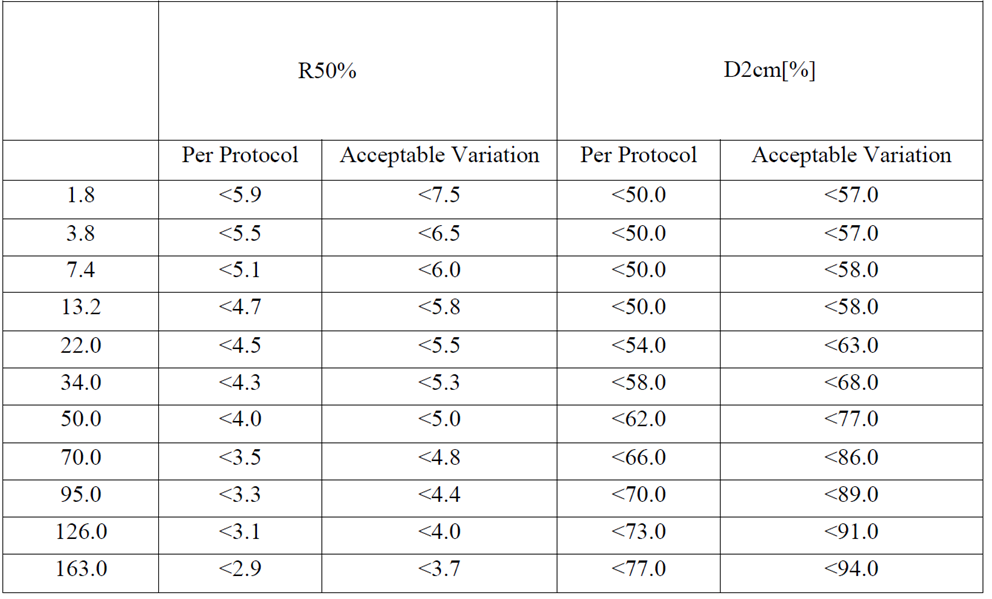


Source: NRG LU002 trial protocol
